# Device-Specific Outcomes of Percutaneous Mechanical Aspiration in Right-Sided Infective Endocarditis: Insights From the Multicenter CLEAR-IE Registry

**DOI:** 10.1016/j.jscai.2026.105494

**Published:** 2026-08-04

**Authors:** Guy Robinson, Kiyan Heybati, Benjamin Hibbert, Sripal Bangalore, Pete Fong, David Zlotnick, Brittany Zwischenberger, Sameh Sayfo, Stephanie Younes, Joseph Ingrassia, Muhammad Hammadah, Anand Prasad, Nadira Hamid, Pedro Villablanca, Amir Kaki, Mohammed Qintar, Marquand Patton, Evin Yucel, Pradeep Yadav, Andrew Klein, Larry Baddour, Paul Sorajja, John Moriarty, Sahil A. Parikh, Sanjum Sethi, Abdallah El Sabbagh

**Affiliations:** a Department of Internal Medicine, Mayo Clinic, Jacksonville, Florida; b Department of Internal Medicine, Mayo Clinic, Rochester, Minnesota; c Department of Cardiovascular Disease, Mayo Clinic, Rochester, Minnesota; d Division of Cardiovascular Disease, New York University School of Medicine, New York, New York; e Division of Cardiovascular Disease, Vanderbilt University Medical Center, Nashville, Tennessee; f Division of Cardiovascular Disease, University at Buffalo, Jacobs School of Medicine and Biomedical Sciences, Buffalo, New York; g Division of Cardiothoracic Surgery, Duke University, Durham, North Carolina; h Division of Cardiovascular Disease, Baylor Scott & White The Heart Hospital − Plano, Plano, Texas; i Division of Cardiovascular Medicine, Promedica Toledo, Toledo, Ohio; j Division of Cardiovascular Disease, Hartford Healthcare Heart and Vascular Institute, Hartford, Connecticut; k Division of Cardiovascular Disease, University of Texas Health San Antonio, San Antonio, Texas; l Department of Cardiovascular Disease, Minneapolis Heart Institute, Minneapolis, Minnesota; m Division of Cardiovascular Disease, Henry Ford Hospital, Detroit, Michigan; n Division of Cardiovascular Disease, Ascension Michigan St. John Hospital, Detroit, Michigan; o Division of Cardiovascular Disease, Sparrow Medical Center, Lansing, Michigan; p Division of Cardiovascular Disease, Palmetto General Hospital, Hialeah, Florida; q Division of Cardiovascular Disease, Massachusetts General Hospital, Boston, Massachusetts; r Division of Cardiovascular Disease, Piedmont Heart Institute, Atlanta, Georgia; s Division of Public Health, Infectious Diseases and Occupational Medicine, Department of Medicine, Mayo Clinic College of Medicine and Science, Rochester, Minnesota; t Department of Radiology and Department of Cardiology, University of California, Los Angeles, Los Angeles, California; u Division of Cardiovascular Disease, NewYork-Presbyterian Hospital/Columbia University Irving Medical Center, New York, New York; v Department of Cardiovascular Disease, Mayo Clinic, Jacksonville, Florida

**Keywords:** aspiration, continuous-flow aspiration, endocarditis, noncontinuous-flow aspiration, patient outcomes

Percutaneous mechanical aspiration (PMA) has emerged as a catheter-based strategy for vegetation debulking in select patients with right-sided infective endocarditis (RSIE).^1^^,^^2^ In the CLEAR-IE (Cardiac Lesion Extraction and Aspiration Registry for Infective Endocarditis) multicenter registry, PMA was associated with high procedural success, with adverse events largely reflecting underlying disease severity.^1^ PMA platforms are categorized by aspiration mechanics into continuous-flow (CF) systems, which use centrifugal pumping with venovenous reinfusion, and noncontinuous-flow (NCF) systems, which rely on intermittent suction without a closed circuit.^2^^,^^3^ Comparative outcomes between these strategies remain undefined. We evaluated PMA outcomes in RSIE from the CLEAR-IE registry, stratified by device type.

CLEAR-IE is a multicenter, retrospective registry encompassing 19 US institutions evaluating outcomes of PMA in RSIE; its design has been reported previously.^1^ Consecutive adult patients meeting the modified Duke criteria who underwent PMA were included, whereas those treated for left-sided IE were excluded. Device selection was at operator discretion. Data were retrospectively collected using a standardized form, with institutional review board approval at each site. Reporting followed Strengthening the Reporting of Observational Studies in Epidemiology (STROBE) guidelines.

Patients were stratified by aspiration platform into CF and NCF groups. Procedural success was defined as a ≥70% reduction in vegetation size or a residual vegetation ≤10 mm on intraprocedural echocardiography, which included transthoracic echocardiography, transesophageal echocardiography, and intracardiac echocardiography, with selection at the preference of the operator. The primary safety end point was defined as a composite of in-hospital mortality, new pulmonary embolism (PE), worsened tricuspid regurgitation, urgent surgical intervention, or blood loss ≥150 mL.

Statistical analyses were performed using R 4.4.2. Data were summarized using descriptive statistics, with continuous variables presented as median (IQR). To account for baseline differences, nearest-neighbor propensity score matching was performed in a 3:1 ratio (CF to NCF) using age, sex, presence of a cardiac implantable electronic device, intravenous drug use, prior percutaneous coronary intervention, and acute PE as matching variables. Associations between device type and outcomes were assessed using logistic regression and reported as odds ratios with 95% CIs. Complete case analysis was utilized due to the relatively limited amount of missing data. For the propensity-matched analysis, patients with missing data for any covariate included in the propensity score model were excluded from the matched cohort. A two-sided *P* value < .05 denoted statistical significance.

This analysis included 238 patients, with a median age of 43 (IQR, 33-61) years, and 44.1% were women. CF-PMA was used in 202 (84.9%) patients and NCF-PMA in 36 (15.1%) patients. NCF-PMA was performed using AlphaVac (AngioDynamics) in 5 (13.9%) cases, Lightning system (Penumbra) in 22 (61.1%), FlowTriever (Inari Medical) in 3 (8.3%), and other/combination in 6 (16.7%). The CF cohort was younger (42.0 [IQR, 32.0-58.0] vs 59.5 [IQR, 40.0-65.0] years; *P* = .004), had a higher prevalence of intravenous drug use (54.0% vs 28.6%; *P* = .006), and fewer cardiac implantable electronic devices (19.7% vs 66.7%; *P* < .001) and cardiovascular comorbidities (Table 1, Supplemental Table S1). Baseline vegetation size was similar (CF: 23.0 [IQR, 19.0-30.0] vs NCF: 26.0 [IQR, 20.0-30.0] mm; *P* = .69), with the CF cohort having fewer lead vegetations (18.3% vs 54.5%; *P* < .001). In patients without lead vegetation, tricuspid valve involvement did not differ between CF and NCF cohorts (61.8% vs 53.8%; *P* = .57). There was no significant difference in days of antibiotic therapy prior to intervention between groups (CF: 8.0 [IQR, 5.0-13.0] vs NCF: 6.0 [IQR, 2.5-12.0] days; *P* = .28). Procedure time was similar (91.0 [IQR, 70.3-122.8] vs 105.0 [IQR, 75.0-140.0] minutes; *P* = .76), while general anesthesia was utilized less in the CF cohort (86.1% vs 100.0%; *P* = 0.017). CF more commonly used transesophageal echocardiography guidance (90.1% vs 72.2%; *P* = .012), with lower use of intracardiac echocardiography (8.9% vs 25.0%; *P* = .012). There was no difference in procedural success between groups (CF: 87.0% vs NCF: 97.1%; *P* = .091). Procedural median blood loss was lower with CF (50 [IQR, 50.0-100.0] vs 150 [IQR, 100.0-250.0] mL; *P* = 0.037), and the primary safety end point occurred less frequently (37.6% vs 63.9%; *P* = .003). In-hospital mortality (CF: 10.0% vs NCF: 8.3%; *P* = .76) and 6-week mortality (CF: 14.4% vs NCF: 11.8%; *P* = .69) were similar.

**Table 1:** Characteristics and outcomes of the non-matched and matched cohorts.

Variable	Non-matched cohort				Matched cohort			
	Continuous aspiration (n = 202)	Non-continuous aspiration (n = 36)	Total (N = 238)	*P* value	Continuous aspiration (n = 78)	Non-continuous aspiration (n = 34)	Total (N = 112)	*P* value
Patient characteristics								
Age at time of procedure, y	42.0 (32.0-58.0)	59.5 (40.0-65.0)	43.0 (33.3-61.0)	.004	46.0 (35.0-66.3)	59.0 (38.0-64.8)	49.0 (35.8-65.0)	.30
Female sex	89 (44.1)	16 (44.4)	105 (44.1)	.97	35 (44.9)	15 (44.1)	50 (44.6)	.94
Hypertension	63 (31.2)	18 (50.0)	81 (34.0)	.028	31 (39.7)	16 (47.1)	47 (42.0)	.47
Hyperlipidemia	38 (18.9)	13 (36.1)	51 (21.5)	.021	19 (24.4)	11 (32.4)	30 (26.8)	.38
Diabetes mellitus	43 (21.3)	9 (25.0)	52 (21.8)	.62	22 (28.2)	7 (20.6)	29 (25.9)	.40
Myocardial infarction	15 (7.4)	7 (19.4)	22 (9.2)	.022	8 (10.3)	6 (17.6)	14 (12.5)	.28
Stroke	14 (6.9)	1 (2.8)	15 (6.3)	.35	7 (9.0)	1 (2.9)	8 (7.1)	.25
Intravenous drug use	109 (54.0)	10 (28.6)	119 (50.2)	.006	37 (47.4)	10 (29.4)	47 (42.0)	.08
Prior PCI	16 (8.0)	8 (22.2)	24 (10.1)	.009	12 (15.4)	7 (20.6)	19 (17.0)	.50
Pacemaker/defibrillator	39 (19.9)	24 (66.7)	63 (27.2)	<.001	34 (43.6)	23 (67.6)	57 (50.9)	.02
Hospitalization/procedure characteristics								
Acute PE	114 (56.4)	11 (30.6)	125 (52.5)	.004	34 (43.6)	10 (29.4)	44 (39.3)	.16
Days on antibiotics prior to intervention	8.0 (5.0-13.0)	6.0 (2.5-12.0)	8.0 (5.0-13.0)	.28	9.0 (5.0-14.0)	5.5 (2.3-12.0)	8.0 (4.0-13.8)	.29
Device lead vegetation	34 (18.3)	18 (54.5)	52 (23.7)	<.001	28 (39.4)	17 (54.8)	45 (44.1)	.015
Longest measured vegetation size, mm	23.0 (19.0-30.0)	26.0 (20.0-30.0)	24.0 (19.0-30.0)	.69	27.0 (20.0-33.3)	25 (19.5-30.0)	26.0 (20.0-30.0)	.75
Procedure time, min	91.0 (70.3-122.8)	105.0 (75.0-140.0)	95.0 (71.5-127.5)	.76	104.0 (73.3-151.5)	104.0 (75.0-125.0)	104.0 (75-140.0)	.19
General anesthesia	174 (86.1)	36 (100.0)	210 (88.2)	.017	67 (85.9)	34 (100.0)	101 (90.2)	.020
Guidance technique								
TEE	182 (90.1)	26 (72.2)	208 (87.4)	.012	71 (91)	24 (70.6)	95 (84.8)	.021
ICE	18 (8.9)	9 (25.0)	27 (11.3)		6 (7.7)	9 (26.5)	15 (13.4)	
TTE	2 (1.0)	1 (2.8)	3 (1.3)		1 (1.3)	1 (2.9)	2 (1.8)	
Outcome								
Procedural success^a^	154 (87.0)	33 (97.1)	187 (88.6)	.091	61 (88.4)	31 (96.9)	92 (91.1)	.17
Adverse events^b^	76 (37.6)	23 (63.9)	99 (41.6)	.003	33 (42.3)	21 (61.8)	54 (48.2)	.05
Blood loss, mL	50.0 (50.0-100.0)	150.0 (100.0-250.0)	100.0 (50.0-150.0)	.037	50.0 (50.0-100.0)	125.0 (100.0-250.0)	100.0 (50.0-200.0)	.008
Blood loss ≥150 mL	29 (19.7)	18 (52.9)	47 (26.0)	<.001	13 (24.1)	16 (50.0)	29 (33.7)	.01
Blood transfusion	95 (47.3)	14 (38.9)	109 (46.0)	.35	40 (51.9)	13 (38.2)	53 (47.7)	.18
Worsening TR	27 (14.7)	5 (13.9)	32 (14.5)	.67	16 (22.9)	5 (14.7)	21 (20.2)	.33
New pulmonary embolism	14 (7.0)	4 (11.1)	18 (7.6)	.39	4 (5.1)	4 (11.8)	8 (7.1)	.21
Death at 6 wk	25 (14.4)	4 (11.8)	29 (13.9)	.69	14 (20.6)	3 (9.4)	17 (17.0)	.16

Values are presented as n (%) or median (IQR) using complete case analysis (see Supplemental Table S1 for missing data).

ICE, intracardiac echocardiogram; PCI, percutaneous coronary intervention; TEE, transesophageal echocardiogram; TR, tricuspid regurgitation; TTE, transthoracic echocardiogram.

a Defined as reduction in vegetation size ≥70% or residual vegetation ≤10 mm.

b Composite of in-hospital death, new pulmonary embolism, worsened tricuspid regurgitation, emergency surgery, and blood loss ≥150 mL.

After propensity matching, 112 patients were included (78 CF-PMA; 34 NCF-PMA). Median age was 49 (IQR, 36-65) years, and 44.6% were women, with similar baseline characteristics. There was no difference in procedural success between groups (CF: 88.4% vs NCF: 96.9%; *P* = .17). The composite of adverse events was lower in the CF group compared with NCF (42.3% vs 61.8%; *P* = .05). CF was associated with fewer cases of blood loss ≥150 mL (24.1% vs 50.0%; *P* = .01) and lower transfusion requirements (1.5 [IQR, 1.0-3.0] vs 2.0 [IQR, 1.5-4.0] units; *P* = .05). Rates of new PE (CF: 5.1% vs NCF: 11.8%; *P* = .21) and worsening tricuspid regurgitation (CF: 22.9% vs NCF: 14.7%; *P* = .33) were similar. There was no difference in in-hospital mortality (CF: 14.1% vs NCF: 5.9%; *P* = .21) or 6-week mortality (CF: 20.6% vs NCF: 9.4%; *P* = .16). On multivariable analysis, CF was independently associated with lower odds of the primary safety end point compared with NCF (adjusted odds ratio, 0.35; 95% CI, 0.14-0.83; *P* = .02).

In this multi-center subanalysis from the CLEAR-IE registry, CF and NCF aspiration systems demonstrated similarly high procedural success for PMA in RSIE. However, NCF-PMA was associated with a higher rate of the primary safety end point, driven predominantly by blood loss.

CF aspiration with the AngioVac system (AngioDynamics) enables uninterrupted high-volume aspiration with venovenous reinfusion, minimizing repeated catheter-vegetation engagement and reducing procedural blood loss. These features may mitigate embolic risk but require greater procedural complexity, including large-bore venous access, specialized personnel, and extracorporeal circuit management, potentially limiting applicability in select settings.^3^, ^4^, ^5^

NCF aspiration systems can offer a simpler single-access procedural setup, but rely on intermittent aspiration without reinfusion, predisposing to greater blood loss and potentially limiting suitability for large vegetations, where repeated catheter-lesion interactions could increase embolic risk and effective debulking may require aspiration of larger blood volumes.

In this analysis, the similar vegetation size between groups suggests that differences in outcomes were not primarily driven by lesion burden. NCF systems were more frequently used in patients with lead-associated vegetations, likely reflecting operator preference for maneuverability in this setting. This selection pattern may explain the similar procedural durations observed, as any setup efficiency with NCF systems may have been offset by the need for concomitant lead extraction.

Adverse events were more frequent with NCF-PMA and were largely attributable to blood loss. This occurred without differences in access-site or vascular bleeding, suggesting that lack of venovenous reinfusion, intrinsic to NCF systems, was the primary driver of blood loss. Additionally, the absence of differential embolic or valvular risk across device platforms supports the procedural safety of PMA and underscores the predominance of disease substrate over device characteristics.

This study is limited by its retrospective registry design, device imbalance favoring CF systems, and lack of standardized procedural protocols or systematic postprocedural imaging. Accordingly, these findings should be considered hypothesis-generating.

In summary, CF and NCF aspiration systems were associated with similar procedural success and comparable rates of embolic and valvular complications in RSIE. Device selection should therefore be guided by vegetation characteristics and operator experience. Prospective studies are needed to further define the relative safety and efficacy of these aspiration strategies.

## CRediT authorship contribution statement

**Guy Robinson:** Writing – review & editing, Writing – original draft, Project administration, Methodology, Investigation, Formal analysis, Conceptualization. **Kiyan Heybati:** Writing – review & editing, Writing – original draft, Visualization, Project administration, Investigation, Formal analysis, Data curation, Conceptualization. **Benjamin Hibbert:** Writing – review & editing, Data curation, Conceptualization. **Sripal Bangalore:** Writing – review & editing, Data curation, Conceptualization. **Pete Fong:** Writing – review & editing, Data curation, Conceptualization. **David Zlotnick:** Writing – review & editing, Data curation, Conceptualization. **Brittany Zwischenberger:** Writing – review & editing, Data curation, Conceptualization. **Sameh Sayfo:** Writing – review & editing, Data curation, Conceptualization. **Stephanie Younes:** Writing – review & editing, Data curation, Conceptualization. **Joseph Ingrassia:** Writing – review & editing, Data curation, Conceptualization. **Muhammad Hammadah:** Writing – review & editing, Data curation, Conceptualization. **Anand Prasad:** Writing – review & editing, Data curation, Conceptualization. **Nadira Hamid:** Writing – review & editing, Data curation, Conceptualization. **Pedro Villablanca:** Writing – review & editing, Data curation, Conceptualization. **Amir Kaki:** Writing – review & editing, Data curation, Conceptualization. **Mohammed Qintar:** Writing – review & editing, Data curation, Conceptualization. **Marquand Patton:** Writing – review & editing, Data curation, Conceptualization. **Evin Yucel:** Writing – review & editing, Data curation, Conceptualization. **Pradeep Yadav:** Writing – review & editing, Data curation, Conceptualization. **Andrew Klein:** Writing – review & editing, Data curation, Conceptualization. **Larry Baddour:** Writing – review & editing, Data curation, Conceptualization. **Paul Sorajja:** Writing – review & editing, Data curation, Conceptualization. **John Moriarty:** Writing – review & editing, Data curation, Conceptualization. **Sahil A. Parikh:** Writing – review & editing, Data curation, Conceptualization. **Sanjum Sethi:** Writing – review & editing, Data curation, Conceptualization. **Abdallah El Sabbagh:** Writing – review & editing, Writing – original draft, Project administration, Methodology, Investigation, Data curation, Conceptualization.

## Peer review statement

Associate Editor Sahil A. Parikh had no involvement in the peer review of this article and has no access to information regarding its peer review. Full responsibility for the editorial process for this article was delegated to Associate Editor Andrew M. Goldsweig.

## Declaration of competing interest

Sripal Bangalore serves on advisory boards for Abbott Vascular, Boston Scientific, Biotronik, Amgen, Pfizer, Merck, REATA, Inari, and Truvic. David Zlotnick serves on speakers bureaus for Abiomed, Inari Medical, and AngioDynamics and is a consultant for AngioDynamics. Sameh Sayfo is a consultant for AngioDynamics, Inari Medical, Penumbra, Imperative Care, and Boston Scientific. Stephanie Younes serves on the speakers bureau for AngioDynamics. Nadira Hamid consults for Abbott Structural, Anteris, AMX, 4C Medical Technologies, Alleviant Medical, Edwards Lifesciences, Philips, GE, Valcare Med Ltd, Vdyne, and W. L. Gore. Pedro Villablanca is a consultant for Edwards Lifesciences, Medtronic, AngioDynamics, and Abiomed. Marquand Patton is a consultant for AngioDynamics. Paul Sorajja consults for 4C Medical, Abbott Structural, Adona, Boston Scientific, ConKay, CroiValve, Cultiv8, Edwards Lifesciences, Egg Medical, EvolutionMed, Foldax, GE Medical, Haemonetics, InQ8, LAZA, Medtronic, Phillips, Polares, W. L. Gore, vDyne, Unorthodox Ventures, Valcare, and xDot and has received institutional research grants from Abbott, Boston Scientific, Edwards Lifesciences, and Medtronic. John Moriarty has received support for the present manuscript from Penumbra; has received consulting fees from AngioDynamics, Innova Vascular, Inquis Medical, Boston Scientific, Imperative Care, and Thrombolex; has received payment or honoraria for lectures, presentations, speaker’s bureaus, manuscript writing, or educational events from AngioDynamics and Penumbra; and has stock or stock options from PAVmed and Thrombolex. Sahil A. Parikh has received institutional grants and research support from Abbott Vascular, Shockwave Medical, TriReme Medical, SurModics, Silk Road Medical, and the National Institutes of Health; has received consulting fees from Terumo and Abiomed; and has served on the advisory boards of Abbott, Medtronic, Boston Scientific, CSI, Janssen, and Philips. Sanjum Sethi has received honoraria from Boston Scientific, Terumo, Inari, Penumbria, Janssen, and Chiesi. Guy Robinson, Kiyan Heybati, Benjamin Hibbert, Pete Fong, Brittany Zwischenberger, Joseph Ingrassia, Muhammad Hammadah, Anand Prasad, Amir Kaki, Mohammed Qintar, Evin Yucel, Pradeep Yadav, Andrew Klein, Larry Baddour, Paul Sorajja, and Abdallah El Sabbagh declare no competing financial, professional, or personal interests that may have influenced the conduct of this study and its presentation in this manuscript.

## Footnotes

To access the supplemental material accompanying this article, visit the online version of the *Journal of the Society for Cardiovascular Angiography & Interventions* at 10.1016/j.jscai.2026.105494.
