# Supplementary material for: Device-Specific Outcomes of Percutaneous Mechanical Aspiration in Right-Sided Infective Endocarditis: Insights From the Multicenter CLEAR-IE Registry
Source: J Soc Cardiovasc Angiogr Interv. 2026 Aug 4;5(9):105494. doi: 10.1016/j.jscai.2026.105494 (PMC13591517; doi:10.1016/j.jscai.2026.105494)
Supplement: Supplementary file 1 [file mmc1.docx]

**ELECTRONIC SUPPLEMENTARY MATERIALS**

**Device-Specific Outcomes of Percutaneous Mechanical Aspiration in Right-Sided Infective Endocarditis: Insights from the Multi-center CLEAR-IE Registry**

Guy Robinson*, MBBS, Kiyan Heybati*, MD, MSc; Benjamin Hibbert, MD, PhD; Sripal Bangalore, MD, MHA; Pete Fong, MD; David Zlotnick, MD; Brittany Zwischenberger, MD; Sameh Sayfo, MD, MBA; Stephanie Younes, MD; Joseph Ingrassia, MD; Muhammad Hammadah, MD; Anand Prasad, MD; Nadira Hamid, MD; Pedro Villablanca, MD, MSc; Amir Kaki, MD; Mohammed Qintar, MD, MSc; Marquand Patton, DO; Evin Yucel, MD; Pradeep Yadav, MD; Andrew Klein, MD; Larry Baddour, MD; Paul Sorajja, MD; John Moriarty, MD; Sahil Parikh, MD; Sanjum Sethi, MD, MPH; Abdallah El Sabbagh, MD

**Contributed equally and serve as co-first authors*

**Table of Contents:**

**Supplementary Table 1.** Missing data in the non-matched and matched cohorts, number of missing observations........................2-3

**Supplementary Table 1.** Missing data in the non-matched and matched cohorts, number of missing observations

|  | **Non-matched Cohort** | | | **Matched Cohort** | | |
| --- | --- | --- | --- | --- | --- | --- |
| **Variable** | **Continuous Aspiration (N=202)** | **Non-Continuous Aspiration (N=36)** | **Total (N=238)** | **Continuous Aspiration (N=78)** | **Non-Continuous Aspiration (N=34)** | **Total (N=112)** |
| ***Patient characteristics*** | | | | | | |
| Age at time of procedure | 1 | 0 | 1 | 0 | 0 | 0 |
| Female sex | 0 | 0 | 0 | 0 | 0 | 0 |
| HTN | 0 | 0 | 0 | 0 | 0 | 0 |
| HLD | 1 | 0 | 1 | 0 | 0 | 0 |
| DM | 0 | 0 | 0 | 0 | 0 | 0 |
| MI | 0 | 0 | 0 | 0 | 0 | 0 |
| Stroke | 0 | 0 | 0 | 0 | 0 | 0 |
| IV drug use | 0 | 1 | 1 | 0 | 0 | 0 |
| Prior PCI | 1 | 0 | 1 | 0 | 0 | 0 |
| PM/Defibrillator | 6 | 0 | 6 | 0 | 0 | 0 |
| ***Hospitalization/procedure characteristics*** | | | | | | |
| Acute PE | 0 | 0 | 0 | 0 | 0 | 0 |
| Days on antibiotics prior to intervention | 15 | 1 | 16 | 6 | 0 | 6 |
| Device lead vegetation | 21 | 4 | 25 | 7 | 3 | 10 |
| Median vegetation size, long, mm | 67 | 3 | 70 | 26 | 3 | 29 |
| Procedure time, minutes | 36 | 3 | 39 | 20 | 3 | 23 |
| General anesthesia | 0 | 0 | 0 | 0 | 0 | 0 |
| Guidance technique | | | | | | |
| TEE | 0 | 0 | 0 | 0 | 0 | 0 |
| ICE | 0 | 0 | 0 | 0 | 0 | 0 |
| TTE | 0 | 0 | 0 | 0 | 0 | 0 |
| ***Outcome*** | | | | | | |
| Procedural success^a^ | 25 | 2 | 27 | 9 | 2 | 11 |
| Adverse events^b^ | 0 | 0 | 0 | 0 | 0 | 0 |
| Blood loss, mL | 55 | 2 | 57 | 24 | 2 | 26 |
| Blood Loss >150 mL | 55 | 2 | 57 | 24 | 2 | 26 |
| Blood Transfusion | 1 | 0 | 1 | 1 | 0 | 1 |
| Worsening TR | 18 | 0 | 18 | 8 | 0 | 8 |
| New PE | 1 | 0 | 1 | 0 | 0 | 0 |
| Death at 6 weeks | 28 | 2 | 30 | 10 | 2 | 12 |

Data presented as N (%) or median (IQR)

**^a^**Defined as reduction in vegetation size >70% or residual vegetation <10 mm

**^b^**Composite of in-hospital death, new pulmonary embolism, worsened tricuspid regurgitation, emergency surgery, and blood loss ≥150 mL.

**Abbreviations:**

**HTN** – hypertension; **HLD** – hyperlipidemia; **DM** – diabetes mellitus; **MI** – myocardial infarction; **PCI** – percutaneous coronary intervention; **PM** – pacemaker; **PE** – pulmonary embolism; **mm** – millimeters, **TEE** – transesophageal echocardiogram; **ICE –** intracardiac echocardiogram; **TTE** – transthoracic echocardiogram; **mL** – milliliters; **TR** – tricuspid regurgitation.
